# Association between Life’s Crucial 9 and metabolic dysfunction-associated steatotic liver disease: the mediating role of neutrophil-percentage-to-albumin ratio

**DOI:** 10.3389/fnut.2025.1549089

**Published:** 2025-06-06

**Authors:** Na Zhu, Yanyan Li, Yingying Lin, XinYu Cui, Xin Li

**Affiliations:** ^1^Center of Integrative Medicine, Beijing Ditan Hospital, Capital Medical University, Beijing, China; ^2^Center of Integrative Medicine, Peking University Ditan Teaching Hospital, Beijing, China

**Keywords:** Life’s Crucial 9, MASLD, liver fibrosis, NPAR, NHANES, mediation analysis

## Abstract

**Background:**

The development of metabolic dysfunction-associated steatotic liver disease (MASLD) is closely associated with cardiovascular health (CVH) status and chronic inflammation. Life’s Crucial 9 (LC9) is the most recent index to assess CVH; its association with MASLD and liver fibrosis is unclear. This study aimed to investigate the association of LC9 with MASLD and hepatic fibrosis and to reveal for the first time the mediating role of a novel inflammatory marker, neutrophil percentage-to-albumin ratio (NPAR), in the association between LC9 and MASLD.

**Methods:**

This study was a cross-sectional analysis of data from the National Health and Nutrition Examination Survey (NHANES) from 2005 to 2018. The United States Fatty Liver Index (US-FLI) ≥ 30 was used to diagnose MASLD, and liver stiffness measurement (LSM) > 8.2 is defined as liver fibrosis. Weighted multifactorial regression, restricted cubic spline analysis (RCS), and subgroup analyses were used to assess the association between LC9 and MASLD and liver fibrosis. Mediation analysis was used to explore the possible mediating role of NPAR in the association of LC9 with MASLD.

**Results:**

A total of 9,623 participants were included in this study. After adjusting for all confounders, LC9 was significantly and negatively associated with both MASLD (OR = 0.59, 95% CI: 0.54–0.64) and hepatic fibrosis (OR = 0.66, 95% CI: 0.45–0.97), with each 10-point increase in the LC9 score decreasing the prevalence by 41% and 34%, respectively. In subgroup analyses, interaction tests showed that age, education, deprivation, obesity, smoking, hypertension, diabetes, and hyperlipidemia significantly affected the association between LC9 and MASLD (P for interaction < 0.05). In addition, NPAR was positively associated with the prevalence of MASLD, with a 5% increase in the prevalence of MASLD for each unit increase in NPAR (OR = 1.05, 95% CI: 1.01–1.09). The positive association between NPAR and MASLD was stronger in younger age groups (<60 years), non-drinkers, and participants without diabetes or hyperlipidemia. Mediation analysis showed that NPAR mediated 2.84% of the association between LC9 and MASLD (*p* < 0.001).

**Conclusion:**

Good CVH status (high LC9 score) was associated with lower prevalence of MASLD and liver fibrosis, and NPAR partially mediated the association between LC9 and MASLD. This study provides new epidemiological evidence for preventing MASLD by improving CVH and inflammatory modulation.

## Introduction

Metabolic dysfunction-associated steatotic liver disease (MASLD), previously termed non-alcoholic fatty liver disease (NAFLD), is the most common chronic liver disease worldwide, affecting approximately 30% of the world’s population. The disease burden of MASLD is increasing with the rising prevalence of obesity, type 2 diabetes, and metabolic syndrome. The pathological process of MASLD progresses from simple steatosis to metabolic dysfunction-associated steatohepatitis (MASH), hepatic fibrosis, cirrhosis, and even hepatocellular carcinoma, posing a serious threat to the health of patients (1, 2). As a hepatic manifestation of metabolic syndrome, MASLD shares several common risk factors with cardiovascular disease (CVD), such as obesity, insulin resistance, hypertension, and dyslipidemia (3). Clinical studies have demonstrated that patients with MASLD have a significantly increased risk of CVD (4–6). Meanwhile, hepatic fibrosis accompanying the progression of MASLD, as a key pathological link in the development of the disease toward the end stage, not only directly affects liver function but also interacts with systemic metabolic disorders and inflammatory responses, further exacerbating the disease deterioration (7). Therefore, a comprehensive assessment of the risk factors associated with the onset and progression of MASLD is essential for early intervention and management of the disease.

In 2022, the American Heart Association (AHA) proposed Life’s Essential 8 (LE8) as a metric for assessing cardiovascular health (CVH), which consists of four health behaviors (healthy diet, physical activity, avoid nicotine exposure, and healthy sleep) and four health factors (weight management, cholesterol control, stable blood glucose levels, and stable blood pressure levels) (8). This assessment model is proposed to provide a quantitative basis for cardiovascular disease risk prediction. In recent years, with the deepening of medical research, the impact of mental health on overall health has gradually become the focus of the academic community. Clinical evidence suggests that psychological disorders such as depression are closely related to pathological processes such as metabolic disorders and inflammatory responses and are independent risk factors for cardiovascular disease (9). The Life’s Crucial 9 (LC9), an emerging comprehensive scoring system, builds on the LE8 by innovatively incorporating mental health dimensions into the assessment, providing a more thorough assessment tool for predicting and preventing cardiovascular disease (10). Several studies have shown that higher LE8 scores are associated with a lower prevalence of MASLD (11–13); Liang et al. showed that LE8 was negatively related to MASLD and advanced liver fibrosis (14). However, the relationship between LC9 and MASLD and liver fibrosis is unclear.

Chronic inflammation plays a central role in the pathological process of MASLD, in which local inflammatory responses in the liver interact with systemic metabolic disturbances to drive the progression of steatosis to steatohepatitis and hepatic fibrosis through the activation of pro-inflammatory signalling pathways and the induction of oxidative stress (15). The neutrophil percentage-to-albumin ratio (NPAR), a novel inflammatory indicator, is significantly associated with NPAR and risk of NAFLD and advanced liver fibrosis (16). Dong et al. found that NPAR levels were positively associated with all-cause mortality and CVD mortality in patients with MASLD (17). In addition, a national representative study in the United States showed that higher levels of NPAR were associated with an increased risk of depression (18), which suggests that NPAR plays a vital role in metabolic diseases and mental health.

Therefore, we hypothesized that LC9 is negatively associated with the risk of developing MASLD and hepatic fibrosis and that NPAR may mediate in the LC9–MASLD association. In this study, we utilized data from the National Health and Nutrition Examination Surveys (NHANES) to verify the above hypotheses and provide a new theoretical basis and research direction for early risk assessment and intervention of MASLD and liver fibrosis.

## Methods

### Study participants

NHANES is an ongoing, nationally representative, cross-sectional survey designed to systematically assess the health and nutritional status of the US population (19). It is administered by the National Center for Health Statistics (NCHS), the NCHS Ethics Review Board approved the study protocol, and all participants provided written informed consent. The NHANES data were made available to the public anonymously, and researchers were not required to apply for ethical review when using the data. The study strictly adhered to the Strengthening the Reporting of Observational Studies in Epidemiology (STROBE) (20) to ensure the standardization, scientificity, and transparency of the reporting of the study results.

**Figure 1:**
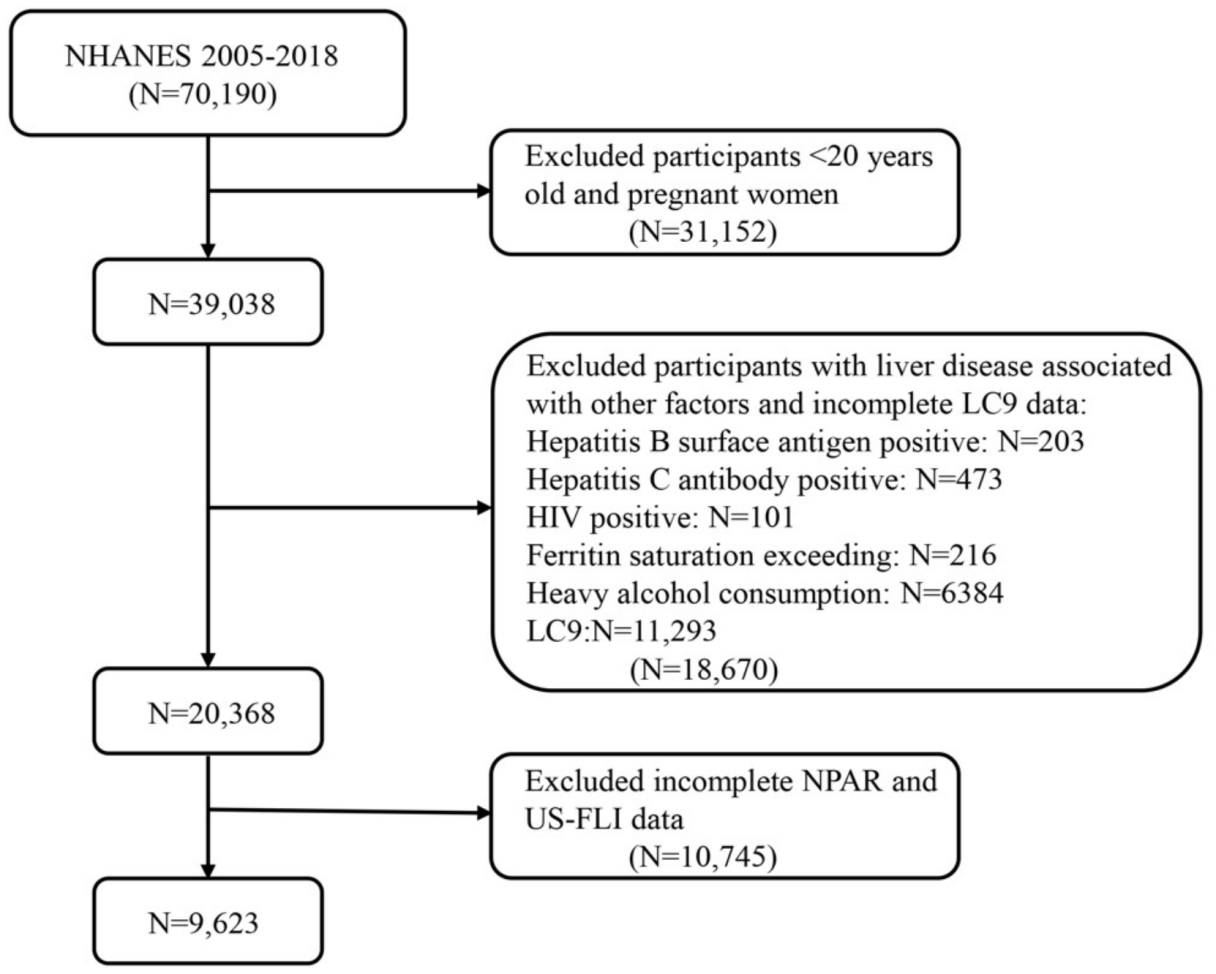
Flow diagram of eligible participant selection in the National Health and Nutrition Examination Survey. MASLD, metabolic dysfunction-associated steatotic liver disease; LC9, Life’s Crucial 9; NPAR, neutrophil percentage-to-albumin ratio.

This study analyzed data from seven NHANES cycles from 2005 to 2018, which included 70,190 participants. After excluding individuals under the age of 20 and pregnant participants, 39,038 participants remained. Subsequently, further exclusions were then applied to those who met any of the following criteria: (1) hepatitis B (*n* = 203); (2) hepatitis C (*n* = 473); (3) HIV-positive (*n* = 101); (4) iron overload (*n* = 216); (5) excessive alcohol consumption (*n* = 6,384) (defined as ≥4 drinks per day for men, ≥3 drinks per day for women or ≥5 drinking days per month); and (6) participants with incomplete NPAR data and US-FLI data (*n* = 10,475). The specific flow is shown in Figure 1. In total, 9,623 adult participants were included in this study.

### Definition of MASLD and liver fibrosis

In this study, we used the United States Fatty Liver Index (USFLI) to define hepatic steatosis. The FLI index is a non-invasive assessment tool developed by CE Ruhl et al. and has been validated in several studies with good sensitivity and specificity (21–23). The calculation of the FLI index requires only basic clinical and laboratory data, including body mass index (BMI), waist circumference, triglycerides (TG), and *γ*-glutamyl transferase. Compared with liver biopsy and other non-invasive methods, FLI is safer, simpler, and less expensive, making it suitable for large-scale population screening and epidemiological studies. In this study, US-FLI ≥ 30 was used as a criterion for diagnosing MASLD after excluding the other liver diseases mentioned above (24). In contrast, liver fibrosis was diagnosed when the LSM value was ≥8.2 kPa (25).

### Measurement of LC9

The LC9 incorporates a depression score based on the LE8, consisting of the following nine components: diet, physical activity, nicotine exposure, sleep health, BMI, lipids, blood glucose, blood pressure, and mental health. Each cardiovascular health (CVH) factor has a standardized score between 0 and 100. The composite LC9 score is calculated as the average of these standardized scores for the nine indicators and reflects an individual’s overall health (10). Dietary indicators are assessed by the Healthy Eating Index (HEI-2015) (26). Physical activity, smoking status, and sleep health were obtained through standardized questionnaires. Trained professionals measured BMI, lipids, blood glucose, and blood pressure. Mental health assessment was obtained from the Patient Health Questionnaire 9 (PHQ-9) (27). Specific calculations for each indicator refer to previous studies, and detailed definitions and scoring methods for the LC9 are provided in the Supplementary Tables S1, S2.

### Assessment of NPAR

In the NHANES database, professional researchers use automated hematological analysis equipment to measure and record the number of neutrophils in blood samples and the serum albumin concentration using the bromocresol purple method. Based on previous studies, NPAR was defined as the neutrophil percentage-to-albumin ratio, and NPAR was calculated according to the following formula: neutrophil percentage (%) × 100/Albumin (g/dL) (18).

### Covariates

Based on previous studies, covariates in this study included age, sex, race, education, marital status, poverty income ratio (PIR), hypertension, diabetes mellitus, and hyperlipidemia. For more information on these covariates, please see Supplementary Table S3.

Standardized questionnaires were used to collect data on age, gender, ethnicity (Mexican American, Non-Hispanic Black, Non-Hispanic White, Other Race), education level (Below high school, High School or above), marital status (Married/Living with partner or not), and the ratio of family income to poverty (Poor: <1.3; Not Poor: ≥1.3). Body measurements, including height and weight, were collected during visits to a mobile examination center (MEC), and body mass index (BMI) was calculated using the formula: weight (kg) / height^2^ (m^2^). Drinking status was categorized into moderate drinking, mild drinking, and never drinking. Smoking status was classified as never smoker (defined as <100 cigarettes in a lifetime), current smoker (defined as ≥100 cigarettes in a lifetime), and former smoker (defined as ≥100 cigarettes and had quit smoking). Hypertension, diabetes, and hyperlipidemia were diagnosed through measurement indicators, prior medication use, and self-reported questionnaire data.

### Statistical analysis

To ensure the accuracy and national representativeness of the analyses, this study considered the NHANES complex sampling design, including sample weights, clustering, and stratification in all statistical analyses. Weights were recalculated for 2005–2018 using “WTMEC2YR” as the weighting variable (new weight = 1/7 × WTMEC2YR). Continuous variables are expressed as mean ± standard deviation; categorical variables are presented as the weighted sample size (percentages). Comparisons of differences between non-MASLD and MASLD groups were analyzed using a weighted Student *t*-test for continuous variables and weighted chi-squared tests for categorical variables.

Weighted multivariate logistic regression was used to explore the association between LC9 and MASLD and liver fibrosis, and weighted linear regression was used to assess the relationship between LC9 and NPAR. To control for confounders as much as possible, the regression model was divided into three levels: Model 1 was not adjusted for any confounders; model 2 adjusted for age, gender, education level, marital status, PIR, and race; and model 3 further adjusted for obesity, smoking status, drinking status, hypertension, diabetes mellitus, and hyperlipidemia based on model 2. The results are presented as odds ratios (OR) or *β* coefficients with 95% confidence intervals (95% CI). Restricted cubic spline regression (RCS) was used to assess the dose–response relationships between LC9 and MASLD, LC9 and liver fibrosis, and NPAR and MASLD.

This study performed subgroup analyses based on the covariates in model 3 to investigate the differences in the relationship between LC9 and MASLD and NPAR and MASLD in different populations. In addition, mediation analyses were performed to assess whether NPAR mediated the effect of LC9 on MASLD occurrence.

All statistical analyses were implemented using the R software (version 4.4.0). The main R packages used were the “survey” package, the “tableone” package, the “rms” package, the “mediation” package, and the “ggplot2” package. Statistical significance was defined as a *p*-value of less than 0.05 on both sides.

## Results

### Baseline characteristics

A total of 9,623 participants were enrolled in this study, and the baseline characteristics of the study population are summarized by the MASLD status categories in Table 1. Study participants were 54% female, predominantly non-Hispanic White (72%), and 33% had MASLD. Compared with non-MASLD participants, those with MASLD were older and had a higher proportion of males, lower educational attainment, higher rates of poverty, higher rates of obesity, and higher rates of metabolism-related disorders (e.g., hypertension, diabetes, and hyperlipidemia). People with MASLD also had lower LC9 scores, HEI-2015 diet scores, and PHQ-9 scores and significantly higher NPAR values.

**Table 1:** Baseline characteristics of all participants were stratified by MASLD, weighted.

Characteristic	Overall, *N* = 43,115,591 (100%)	Non-MASLD, *N* = 28,803,022 (67%)	MASLD, *N* = 14,312,569 (33%)	*p*-value
No. of participants in the sample	9,623	6,367	3,256	**–**
Age (%)				**<0.001**
20–40	12,601,391 (29%)	9,877,653 (34%)	2,723,737 (19%)	
41–60	16,486,021 (38%)	10,705,082 (37%)	5,780,939 (40%)	
>60	14,028,179 (33%)	8,220,287 (29%)	5,807,893 (41%)	
Sex (%)				**<0.001**
Female	23,120,488 (54%)	16,451,140 (57%)	6,669,348 (47%)	
Male	19,995,103 (46%)	12,351,882 (43%)	7,643,221 (53%)	
Race (%)				**<0.001**
Non-Hispanic White	31,079,023 (72%)	20,396,903 (71%)	10,682,120 (75%)	
Non-Hispanic Black	4,271,829 (9.9%)	3,438,119 (12%)	833,710 (5.8%)	
Other	5,062,740 (12%)	3,578,146 (12%)	1,484,594 (10%)	
Mexican American	2,701,999 (6.3%)	1,389,854 (4.8%)	1,312,144 (9.2%)	
Married/live with partner (%)				**0.114**
No	14,220,002 (33%)	9,738,752 (34%)	4,481,249 (31%)	
Yes	28,886,054 (67%)	19,064,270 (66%)	9,821,785 (69%)	
Education level (%)				**<0.001**
Below high school	5,921,388 (14%)	3,338,243 (12%)	2,583,145 (18%)	
High school or above	37,183,981 (86%)	25,455,890 (88%)	11,728,091 (82%)	
PIR (%)				**<0.001**
Poor	7,199,888 (18%)	4,536,278 (17%)	2,663,610 (20%)	
Not poor	33,357,918 (82%)	22,534,332 (83%)	10,823,586 (80%)	
Obesity (%)				**<0.001**
No	27,217,107 (63%)	23,186,896 (81%)	4,030,211 (28%)	
Yes	15,898,484 (37%)	5,616,126 (19%)	10,282,359 (72%)	
Smoking (%)				**<0.001**
Never	25,062,227 (58%)	17,550,126 (61%)	7,512,101 (52%)	
Former	12,047,802 (28%)	7,150,466 (25%)	4,897,336 (34%)	
Current	6,005,562 (14%)	4,102,430 (14%)	1,903,133 (13%)	
Drinking (%)				**<0.001**
Never	5,385,756 (13%)	3,578,953 (13%)	1,806,803 (13%)	
Former	7,165,716 (17%)	4,102,767 (15%)	3,062,949 (22%)	
Mild	20,274,501 (49%)	13,893,516 (49%)	6,380,985 (47%)	
Moderate	8,938,342 (21%)	6,498,398 (23%)	2,439,944 (18%)	
Hypertension (%)				**<0.001**
No	25,256,342 (59%)	19,618,226 (68%)	5,638,116 (39%)	
Yes	17,859,249 (41%)	9,184,796 (32%)	8,674,453 (61%)	
Diabetes (%)				**<0.001**
No	35,984,078 (83%)	26,311,528 (91%)	9,672,550 (68%)	
Yes	7,131,513 (17%)	2,491,494 (8.7%)	4,640,020 (32%)	
Hyperlipidemia (%)				**<0.001**
No	11,173,700 (26%)	9,566,627 (33%)	1,607,073 (11%)	
Yes	31,941,891 (74%)	19,236,395 (67%)	12,705,496 (89%)	
Mean LC9 score (mean (SD))	71.22 (13.64)	75.41 (12.45)	62.79 (11.92)	**<0.001**
LC9, Tertile (%)				**<0.001**
T1	14,407,462 (33%)	6,354,684 (22%)	8,052,778 (56%)	
T2	14,362,207 (33%)	9,300,651 (32%)	5,061,556 (35%)	
T3	14,345,922 (33%)	13,147,687 (46%)	1,198,235 (8.4%)	
Mean psychological health score (mean (SD))	90.50 (21.79)	91.86 (20.02)	87.77 (24.76)	**<0.001**
Mean HEI-2015 diet score (mean (SD))	40.83 (31.74)	43.54 (32.05)	35.39 (30.39)	**<0.001**
Mean physical activity score (mean (SD))	70.62 (41.55)	73.78 (39.82)	64.28 (44.15)	**<0.001**
Mean tobacco exposure score (mean (SD))	76.14 (35.08)	76.60 (35.60)	75.22 (34.01)	**<0.001**
Mean sleep health score (mean (SD))	84.08 (23.93)	85.15 (23.18)	81.93 (25.23)	**<0.001**
Mean body mass index score (mean (SD))	60.80 (33.64)	74.08 (28.00)	34.07 (27.58)	**<0.001**
Mean blood lipid score (mean (SD))	64.21 (29.89)	67.91 (29.58)	56.77 (29.13)	**<0.001**
Mean blood glucose score (mean (SD))	85.43 (24.78)	91.62 (19.32)	72.96 (29.43)	**<0.001**
Mean blood pressure score (mean (SD))	68.40 (31.47)	74.19 (30.28)	56.73 (30.56)	**<0.001**
NPAR (mean (SD))	13.74 (2.52)	13.46 (2.50)	14.31 (2.48)	**<0.001**
NPAR, Tertile (%)				**<0.001**
T1	14,385,766 (33%)	10,916,831 (38%)	3,468,934 (24%)	
T2	14,341,828 (33%)	9,645,147 (33%)	4,696,681 (33%)	
T3	14,387,997 (33%)	8,241,043 (29%)	6,146,954 (43%)	

Mean (SD) for continuous variables: the *p*-value was calculated by the weighted Student *t*-test. Weighted sample size (percentages) for categorical variables: the *p*-value was calculated by the weighted chi-squared test. MASLD, metabolic dysfunction-associated steatotic liver disease; LC9, Life’s Crucial 9; NPAR, neutrophil percentage-to-albumin ratio; PIR, poverty income ratio. Bold values indicate *p* < 0.05.

### Association of LC9 with MASLD and liver fibrosis

**Figure 2:**
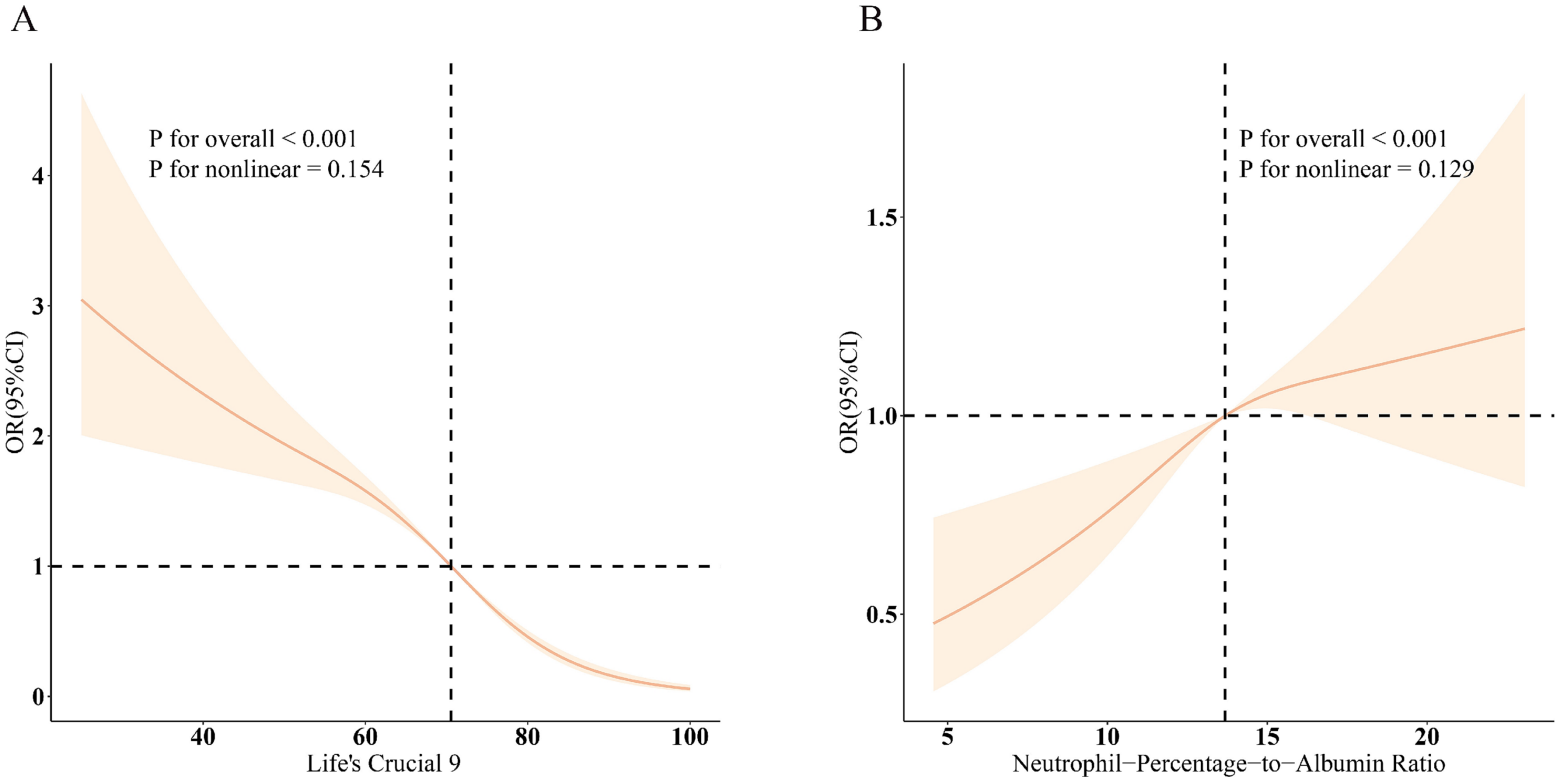
Dose–response relationships between LC9, NPAR, and MASLD. **(A)** LC9–MASLD; **(B)** NPAR–MASLD. OR (solid lines) and 95% confidence levels (shaded areas) were adjusted for age, sex, education level, marital status, PIR, race, obesity, smoking, drinking, hypertension, diabetes, and hyperlipidemia.

**Figure 3:**
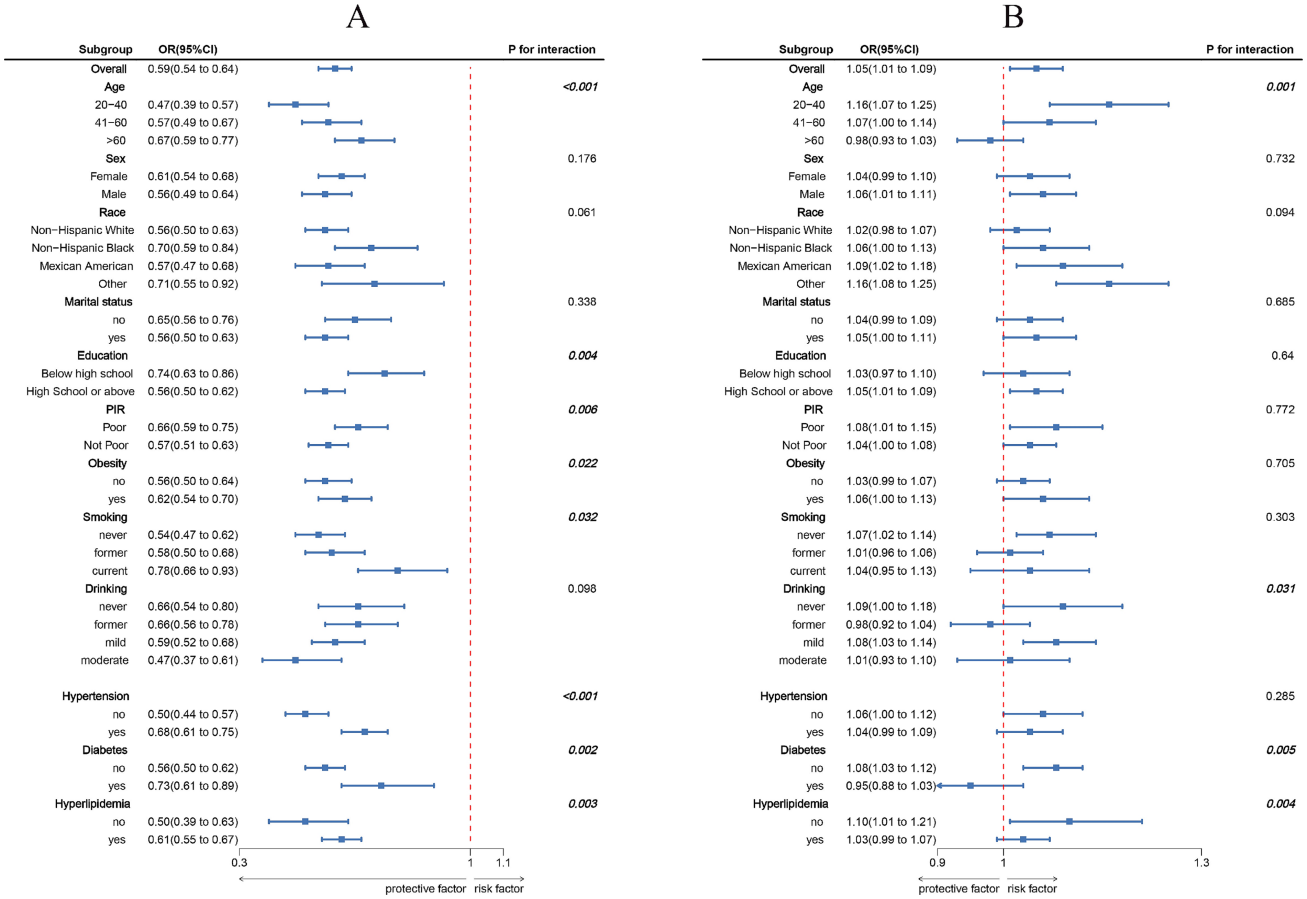
Subgroup analysis between LC9, NPAR, and MASLD. **(A)** LC9–MASLD; **(B)** NPAR–MASLD. ORs were calculated per 10-unit increase in LC9, and each standard deviation increased in NPAR. Analyses were adjusted for age, sex, education level, marital status, PIR, race, obesity, smoking, drinking, hypertension, diabetes, and hyperlipidemia.

The association between LC9 and MASLD and liver fibrosis was analyzed using weighted logistic regression, and the results in Table 2 show a significant negative association between LC9 and MASLD prevalence. After adjusting for all confounding variables, an increase of 10 points per LC9 was associated with a 41% reduction in the prevalence of MASLD (OR = 0.59, 95% CI (0.54, 0.64), *p* < 0.001). Compared with the lowest LC9 tertile, the second tertile adjusted OR was 0.65 (95% CI (0.54, 0.79), *p* < 0.001), and the third tertile adjusted OR was 0.25 (95% CI (0.19, 0.34), *p* < 0.001). Higher LC9 scores were significantly associated with reduced MASLD prevalence (P for trend<0.001). Figure 2A shows the results of the RCS, revealing a significant negative association between the LC9 score and MASLD risk. Subgroup analysis in Figure 3A showed that the LC9 score was negatively associated with MASLD prevalence in all subgroups. Interaction tests showed that age, education, PIR, obesity, smoking, hypertension, diabetes, and hyperlipidemia significantly affected the correlation between LC9 score and MASLD (P for interaction <0.05).

**Table 2:** Association between LC9, NPAR, and MASLD.

Characteristics	Model 1 [OR (95% CI)]	*p*-value	Model 2 [OR (95% CI)]	*p*-value	Model 3 [OR (95% CI)]	*p*-value
LC9–MASLD						
Continuous (per 10 scores)	0.45 (0.43, 0.48)	<0.001	0.42 (0.39, 0.45)	<0.001	0.59 (0.54, 0.64)	<0.001
Tertile						
T1	1 (ref.)		1 (ref.)		1 (ref.)	
T2	0.43 (0.37, 0.50)	<0.001	0.39 (0.33, 0.46)	<0.001	0.65 (0.54, 0.79)	<0.001
T3	0.07 (0.06, 0.09)	<0.001	0.07 (0.05, 0.08)	<0.001	0.25 (0.19, 0.34)	<0.001
P for trend	<0.001		<0.001		<0.001	
NPAR–MASLD						
Continuous	1.15 (1.12, 1.18)	<0.001	1.13 (1.10, 1.17)	<0.001	1.05 (1.01, 1.09)	0.020
Tertile						
T1	1 (ref.)		1 (ref.)		1 (ref.)	
T2	1.53 (1.31, 1.80)	<0.001	1.48 (1.25, 1.75)	<0.001	1.19 (0.99, 1.43)	0.070
T3	2.35 (1.99, 2.77)	<0.001	2.23 (1.87, 2.67)	<0.001	1.48 (1.18, 1.86)	<0.001
P for trend	<0.001		<0.001		<0.001	

Model 1: no covariates were adjusted.

Model 2: age, sex, education level, marital status, PIR, and race were adjusted.

Model 3: age, sex, education level, marital status, PIR, race, obesity, smoking, drinking, hypertension, diabetes, and hyperlipidemia were adjusted.

MASLD, metabolic dysfunction-associated steatotic liver disease; NPAR, neutrophil percentage-to-albumin ratio; PIR, ratio of family income to poverty; OR, odds ratio; CI, confidence interval.

For liver fibrosis, the results in Supplementary Table S4 showed a significant negative association between LC9 and liver fibrosis, with a 34% reduction in the likelihood of developing liver fibrosis for every 10-point increase in LC9 after adjusting for all confounding variables (OR = 0.66, 95% CI (0.45, 0.97), *p* = 0.030). Compared with the lowest tertile of LC9 scores, the adjusted OR for the second tertile was 0.52 (95% CI (0.26, 1.06), *p* = 0.070), and for the third tertile was 0.17 (95% CI (0.04, 0.68), *p* = 0.020). Higher LC9 scores were associated with a lower prevalence of MASLD (trend *p* = 0.010). As shown in Supplementary Figure S1, the RCS results revealed a significant negative correlation between the LC9 score and the risk of liver fibrosis.

### The association between NPAR and MASLD

Table 2 illustrates the association between NPAR and MASLD. After adjusting the model for all confounding variables, a significant positive association between NPAR and the prevalence of MASLD was found. Each unit increase in NPAR was associated with a 5% increase in MASLD prevalence (OR = 1.05, 95% CI (1.01, 1.09), *p* = 0.02). Compared with the lowest NPAR tertile, the second tertile adjusted OR increased from 1.19 (95% CI (0.99, 1.43), *p* = 0.070) to 1.48 (95% CI (1.18, 1.86), *p* = 0.070) in the third tertile, with a 48% increase in MASLD prevalence. Higher NPAR was significantly associated with increased MASLD prevalence (P for trend<0.001).

Figure 2B shows a significant positive association between NPAR and MASLD. Figure 3 shows the results of the subgroup analyses; the positive correlation between NPAR and the risk of MASLD was stronger in participants who were younger than 60 years of age, who had never consumed alcohol, who consumed small amounts of alcohol, and who did not have diabetes mellitus or hyperlipidemia.

### The association between LC9 and NPAR

Table 3 shows the association between LC9 and NPAR, which was statistically significant after adjusting for all covariates (*β* = −0.38, 95% CI (−0.44, −0.31), *p* < 0.001).

**Table 3:** Association between LC9 and NPAR.

Characteristic	*β*	95%CI	*p*-value
LC9–NPAR	−0.38	(−0.44, −0.31)	<0.001

Adjusted for age, sex, education level, marital status, PIR, race, obesity, smoking, drinking, hypertension, diabetes, and hyperlipidemia.

### Mediating role of NPAR in the association of LC9 and MASLD

**Figure 4:**
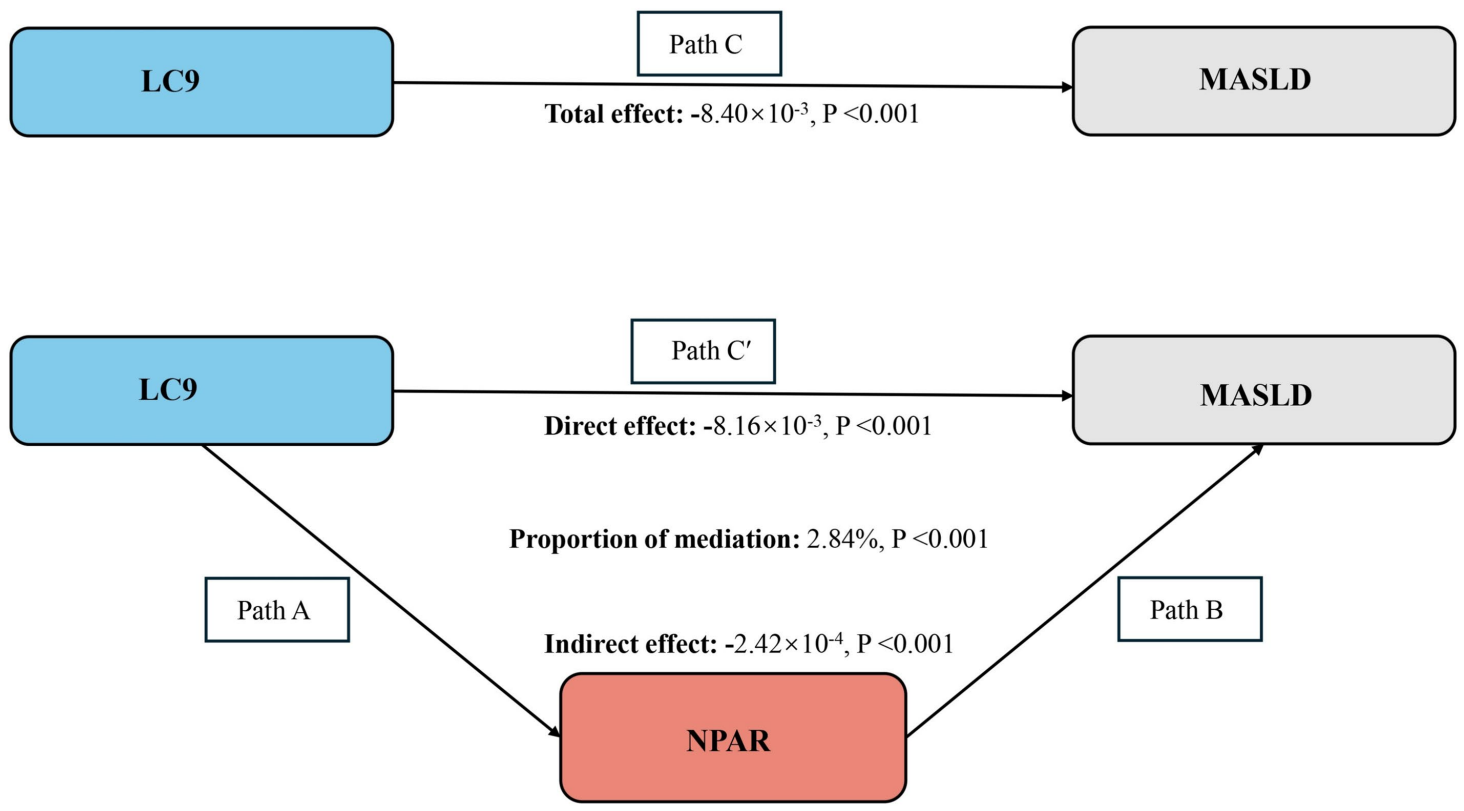
Schematic diagram of the mediation effect analysis. Path C indicates the total effect; path C′ indicates the direct effect. The indirect effect is estimated as the multiplication of paths A and B (path A*B). The mediated proportion is calculated as indirect effect/(indirect effect + direct effect) × 100%. MASLD, metabolic dysfunction-associated steatotic liver disease; LC9, Life’s Crucial 9; NPAR, neutrophil percentage-to-albumin ratio. Analyses were adjusted for age, sex, education level, marital status, PIR, race, obesity, smoking, drinking, hypertension, diabetes, and hyperlipidemia.

Our study fulfilled the prerequisites for conducting mediation analyses based on the above analyses. As shown in Figure 4, after adjusting for all covariates, we observed a mediating effect of NPAR. The indirect impact of NPAR = −2.42*10^−4^, *p* < 0.001 and direct effect = −8.16*10^−3^, *p* = 0.036 mediates 2.84% of the correlation between the LC9 score and MASLD.

## Discussion

In this nationally representative study of US adults, we demonstrated for the first time that the most recent CVH indicator, the LC9, was significantly and negatively associated with both MASLD and hepatic fibrosis; a 10-point increase in LC9 score was associated with a 41% reduction in the prevalence of MASLD and a 34% reduction in the prevalence of hepatic fibrosis. Subgroup analyses showed that age, education, PIR, obesity, smoking, hypertension, diabetes, and hyperlipidemia significantly moderated the strength of the association between LC9 score and MASLD (interaction test *p* < 0.05). In addition, NPAR, a novel marker of inflammatory response, was significantly and positively associated with MASLD, and this association was more prominent in younger age groups (<60 years), non-drinkers, and individuals without diabetes or hyperlipidemia. Notably, NPAR played an important mediating role between LC9 and MASLD, suggesting that elevated LC9 scores may indirectly reduce the risk of MASLD development by modulating the inflammatory state.

Our findings showed a significant negative association between the latest CVH metric, LC9, and the prevalence of MASLD and liver fibrosis, consistent with several previous studies’ findings. A cross-sectional study of the U.S. population found that adults with higher CVH indicators assessed by the LE8 score had a lower risk of developing MAFLD and advanced liver fibrosis (14). An extensive cohort study in China demonstrated that an ideal cardiovascular health baseline and cumulative exposure levels were significantly associated with a reduced risk of NAFLD development and an increased likelihood of regression (28). A prospective analysis in the UK Biobank found that a good lifestyle and better CVH assessed by LE8 were significantly associated with a lower risk of new-onset severe NAFLD (29). The Life’s LC9 cardiovascular health scoring system based on a comprehensive mental health dimension was significantly and negatively associated with MASLD and its progression to liver fibrosis.

Neutrophil percentage-to-albumin ratio (NPAR) is a novel inflammatory marker integrating neutrophil percentage and peripheral blood albumin levels. Elevated neutrophil percentage implies activation of the innate immune system, which plays a vital role in mediating the inflammatory response, while albumin exerts anti-inflammatory and antioxidant effects (30). Therefore, NPAR is a more comprehensive assessment of inflammation than a single marker. A national study in the United States found that a per-unit increase in NPAR was significantly associated with an increased risk of developing NAFLD (16). In addition, a recent study showed that NPAR has good predictive efficacy for all-cause mortality and CVD mortality in patients with MASLD (17). In our research, NPAR was also significantly positively correlated with the prevalence of MASLD, further validating the previous findings. The present study innovatively revealed that NPAR may be a key mediator in regulating the negative association between LC9 and MASLD. This finding not only expands the existing knowledge but also suggests that chronic inflammation plays an important role in the progression of MASLD and its interaction with CVD, which provides a new perspective for understanding the pathological mechanisms of metabolic liver disease.

The pathogenesis of MASLD is complex and involves multifactorial interactions such as obesity, insulin resistance, chronic inflammation, oxidative stress, and lipid metabolism disorders (28). The health behaviors and factors included in LC9 scores may influence the onset and progression of MASLD by improving systemic levels of inflammation, enhancing insulin sensitivity, and reducing fat accumulation. Healthy dietary patterns, such as the Mediterranean diet, are prized for its richness in whole grains, fruits, vegetables, legumes, and healthy fats, and whose anti-inflammatory and antioxidant properties are effective in reducing liver fat deposits and improving insulin sensitivity (31). A very-low-calorie ketogenic diet (VLCKD) also improves hepatic steatosis and hepatic fibrosis by reducing systemic and hepatic hypo-inflammation, thereby reducing hepatic steatosis and hepatic fibrosis (32). Studies have shown that aerobic exercise reduces intrahepatic fat by increasing fat oxidation and improving insulin sensitivity. Resistance exercise increases muscle mass, improves muscle uptake and utilization of glucose, and reduces liver burden (33). Avoiding smoking reduces oxidative stress and inflammatory responses (34). Good sleep helps maintain normal metabolic function and improves insulin sensitivity, which is essential for maintaining a healthy weight and stabilizing metabolic status (35). Obesity is one of the significant risk factors for MASLD. Inflammatory cytokines secreted by adipose tissue under obesity trigger systemic inflammation, leading to insulin resistance, which further contributes to hepatic fat deposition and exacerbates the condition of MASLD (36). Vilar-Gomez et al. showed that a target weight loss of 7–10% effectively reduced lipid accumulation, increased metabolic flexibility, and improved insulin resistance (37). Appropriate non-HDL cholesterol levels, blood pressure, and blood glucose levels may reduce oxidative stress and inflammatory responses, improve insulin resistance, and reduce the risk of MASLD. Depression may lead to immune-mediated destruction of pancreatic *β*-cells, resulting in insulin resistance and diabetes (38). In addition, it has been shown that the prevalence of liver fibrosis and steatosis is significantly higher in the population of patients with type 2 diabetes mellitus (39). Based on the above pathomechanism, it is scientifically plausible that there is a significant correlation between the LC9 score and the prevalence of MASLD and advanced hepatic fibrosis.

Notably, subgroup analyses showed that age, education, PIR, obesity, smoking, and hypertension, diabetes, and hyperlipidemia significantly moderated the strength of the association between LC9 score and MASLD (P for interaction < 0.05). This difference may be due to the differences in health behaviors, medical resources, and disease susceptibility: Young people are more sensitive to health interventions, and highly educated people are more aware of health management, whereas poor people have limited living and medical conditions, which weaken the protective effect of the LC9; obesity, smoking, and metabolic disease patients have reduced preventive efficacy of the LC9 score due to inflammation and metabolic disorders (40). The positive association between NPAR and MASLD was more pronounced in individuals <60 years of age, non-alcohol drinkers, and non-diabetic/hyperlipidemic individuals. The positive association between NPAR and MASLD is more pronounced in individuals <60, non-drinkers, and non-diabetic/hyperlipidemic individuals. The predictive value of NPAR is more prominent in the younger age group, which is metabolically active (41), where the effect of inflammation on hepatic lipid metabolism is likely to be more direct. The association may be masked by complex metabolic disorders in people with comorbid metabolic diseases.

The major strength of this study is the use of a nationally representative sample of US adults to explore for the first time the association of LC9 with the prevalence of MASLD and liver fibrosis. In addition, through mediation analysis, this study revealed the mediating effect of NPAR between LC9 and MASLD, which further enriches our understanding of the mechanisms of MASLD. LC9 is a comprehensive indicator of CVH and provides a new tool for universal health management. NPAR, as an inflammatory marker, can effectively complement the traditional metabolic risk assessment system. These findings provide a solid theoretical basis for developing MASLD prevention strategies.

There are some limitations to this study. First, the non-invasive USFLI score used in this study as a diagnostic tool for hepatic steatosis is not as accurate as liver biopsy, which may lead to misclassification of disease prevalence and, consequently, underestimation or overestimation of the actual risk level of MASLD. Second, the CVH behavioral indicator assessment relied on self-report questionnaires, which may be subject to some measurement error that may affect the accuracy of the study results. Third, although we have adjusted for a variety of potential confounders, there may still be some unmeasured or uncontrolled variables that may have some impact on the study results, thus affecting the generalizability of the findings. Finally, the limitations of the cross-sectional design of this study prevented us from making causal inferences, and further longitudinal studies are needed in the future to investigate the relationship between LC9 scores, NPAR, and MASLD.

## Conclusion

In conclusion, this study shows a significant negative association between LC9 and the prevalence of MASLD and liver fibrosis. NPAR mediates this LC9–MASLD association. This suggests that improving cardiovascular health effectively reduces the risk of MASLD by modulating chronic inflammation and that a comprehensive strategy combining enhanced cardiovascular health with anti-inflammation is an essential public health measure for the prevention and management of MASLD.

## Data Availability

Publicly available datasets were analyzed in this study. This data can be found here: https://wwwn.cdc.gov/nchs/nhanes/Default.aspx.
